# Gilts prefer an open pen to a stall

**DOI:** 10.1038/s41598-024-60617-2

**Published:** 2024-04-27

**Authors:** Thomas Ede, Mia Ceribelli, Thomas D. Parsons

**Affiliations:** Swine Teaching and Research Center, University of Pennsylvania School of Veterinary Medicine, Kennett Square, PA USA

**Keywords:** Pig, Affective state, Personality, Welfare, Crate, Stall, Sow housing, Zoology, Animal behaviour

## Abstract

Stalls or crates are a very common type of housing used on pig farms that restrict an animal’s movement. How this confinement impacts the animal’s affective states is seldom investigated. We conducted a preference test over 7 days where trios of gilts (n = 10 trios, 27.4 ± 1.5 weeks old) had free access between individual self-locking stalls (~ 1.2 m^2^) and a shared open area allowing 2.8 m^2^/animal (71% of total area). Gilts had access to ad libitum feed and water both inside the crates and in the open area. After 7 days, personality traits of the animals were assessed with open field (OF) and novel object (NO) tests. Principal Component Analysis (PCA) yielded two main components, which we defined as *Passivity* and *Engagement*. The median time spent outside the crate was 95.2% as 21/29 of the gilts exhibited a significant preference for pen over crate during the 7-day trial (p < 0.05). Passivity had no relationship with time spent in the open area, but engagement during OF/NO was associated with less use of the open area (OR = 0.39, 95CI = [0.25, 0.60]). Interestingly, gilts were likely to spend less time in the open area at nighttime compared to daytime (Odds Ratio = 0.49, 95CI = [0.40, 0.60]), as well as experimental days passed (OR = 0.70, 95CI = [0.66, 0.73]). During the first daytime and nighttime, 1/29 and 2/29 animals preferred the crate respectively, whereas by the last daytime and nighttime 5 and 9 gilts preferred the crate respectively (p < 0.05). While both intrinsic (personality) and extrinsic (time of day, experimental day) factors appear to influence the gilt’s housing preferences, most gilts significantly prefer an open area to a crate when free access is provided between the two. A smaller subpopulation of animals developed a preference for stalls but still utilize both the stall and the pen throughout the day.

## Introduction

Swine often are housed in confined environments where movement is restricted. A common practice is the use of crates (or stalls) during mating, gestation, and farrowing. Stalls are used to minimize space requirements, reduce the unwanted effects of inter-animal aggression, facilitate individual animal care, and minimize piglet mortality. Most crates are approximately 0.8 m by 2.0 m and prohibit many natural behaviors such as turning around or appropriate social contact. Unsurprisingly, the use of stalls has been questioned. For example in the EU, pregnant sows must be kept in groups instead of individual stalls since 2013^1^. Gestation crates where mother sows spend the majority of their time are being banned, phased out or limited to short-term use in many other countries including the United Kingdom, Sweden, New Zealand, Australia, and Canada. However, crates remain the most common form of sow housing world-wide as their use is still extensive in the United States, and parts of South America and Asia.

Confinement and space allowance has drawn considerable scientific attention for the past few decades, with an increasing number of studies on space allowance in recent years^2^. So far, the welfare impact of confinement has largely focused on physiological, reproductive, and behavioral markers. These markers can be limited in their sensitivity and are usually altered only in the most severe cases^3,4^. For example, no difference in hair cortisol was found between sows housed in crates or loose-housing, with the authors noting the limits of hair cortisol as a measure of chronic stress rather than a similarity between treatments^5^.

As previously noted in a systematic review on sow housing, the assessment of psychological or affective states is a critical gap in the literature^6^. Preference tests (i.e. looking at an animal’ decision when presented with multiple options) constitute a simple way to assess the value animals attach to resources^7^, and allows minimal experimental intervention granting animals more agency over their choices. Preference tests have been used to study pig housing, comparing the use of dry vs wet floor^8^, floor temperatures^9,10^, floor types^11,12^, illuminance^13^, and enrichments^14^. When presented with a choice between confinement in a crate for 30 min or 240 min, most gilts favored the shorter restriction^15^. Sows also showed a preference for an unlocked over a locked crate^16^, and were found to prefer wider crates^17^.

In this study, we aimed to measure the preference of gilts between crates and an open area where social contact was possible and resources were balanced, as well as examine intrinsic and extrinsic factors that might impact their preferences. We predicted that gilts would display a preference for the open area over stalls. We also explored whether gilt preference for an open area would vary based on experimental day, time of day as well as the role of personality traits.

## Methods

All experimental animal procedures were approved by the University of Pennsylvania’s Institutional Animal Care and Use Committee (Protocol #804656), were performed in accordance with our IACUC guidelines and regulations and are reported in accordance with the ARRIVE guidelines. This study was conducted between August 2022 and December 2022 at the Penn Vet Swine and Research Center in Kennett Square, Pennsylvania, USA.

### Animals

To avoid the influence of prior experience with breeding crates, only nulliparous animals that had recently entered the herd were included in the study. Thirty Large white x Landrace gilts (T70 sow line, Norsvin/Topigs, USA) were enrolled at (27.4 ± 1.5 weeks old) in trios (n = 10) of the same age. All trios were familiar, having been housed together in small pens for at least 2 weeks prior to the trial. One gilt became lame during the experiment and was treated with injectable analgesia (flunixin meglumine injection, 50 mg/mL, Norbrook, UK). Her condition did not improve, and this gilt was not included in the analysis.

### Preference

**Figure 1:**
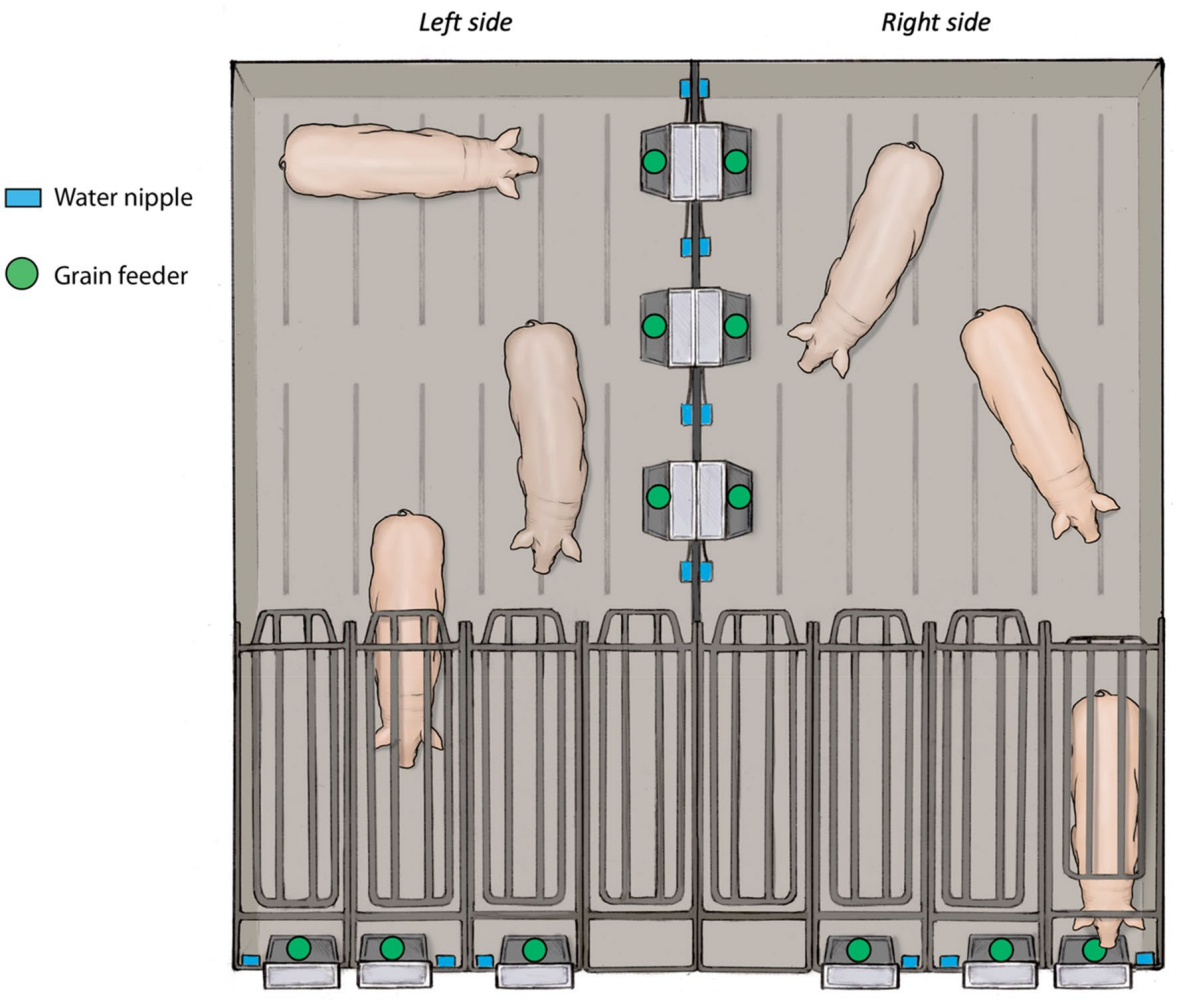
Experimental apparatus. Trios of gilts had free access between an open area and crates for a week. Feed and water were provided ad libitum inside and outside of crates. The 4th crate without a feeder in each pen was locked and not available to animals. Illustration by Ann Sanderson.

On enrollment day, two trios were moved to the experimental pens (Fig. 1) at approximately 14h00. Each experimental pen consisted of three crates (1.9 × 0.6 m, *Schauer Self-Catching Gestation Stall, Prambachkirchen, Austria*) and a common open area (3.1 × 2.7 m, 71% of floor space), all over a slatted concrete floor. The self-catching or free-access stall allowed the gilt to control her use of either the crate or the open pen. Each individual crate was mounted with an individual water nipple and an ad libitum grain feeder (Intak Feeder, Automated Production, Assumption, Illinois, USA), positioned at 45 cm height above the floor. The feed provided was primarily composed of corn and soybean meal (Kalmbach P200NGM 12% protein Natural Sow Gestation Meal, Chambersburg, Pennsylvania, USA). As satiety affects sows’ motivation to leave a crate^18^, we provided ad libitum feeders both in and out of crates. In the open area, three similar grain feeders and water dispensers were provided. Before moving gilts into the pen, a small amount of grain (~ 40 g) was dispensed from all feeders in their respective troughs. Gilts were initially moved into the crates and locked in for a few minutes, allowing experimenters to spray paint their backs and flanks with identifying numbers. The crates were then unlocked, but not opened, by the experimenter and the animals were left in the pen for the next 7 days. Every day at approximately 14h00, all feeders were manually refilled with feed. Cameras positioned over the pens continuously recorded gilts positions over the 7 days.

Gilt position was coded using focal instantaneous sampling^19^ by the location of each gilt once per hour with the coding software BORIS^20^. “Out” was defined by 2 or more limbs outside a crate and “In” required 3 or more limbs in a crate, with gate open or closed.

A subset of the video data was identified to determine quality control metrics related to video coding. Fifty video images captured on the hour were pseudo-randomly selected across all videos and yielded 150 observations (50 images × 3 animals per image). These images were balanced for time of observation (day vs night), day (1 to 7), and side (left vs right). Inter-rater reliability between two observers (MC and TE) was calculated over these 150 observations by Cohen’s Kappa^21^ and found to be excellent (K = 0.84). To ensure our sampling frequency was adequate, we compared instantaneous sampling rates of every 15 min versus every 60 min. For each hourly scan, corresponding sub-samples were conducted every 15 min (e.g. 10h00, 10h15, 10h30 and 10h45), resulting in a total of 600 observations (50 initial scans × 3 individuals × 4 scans within the hour). We compared the distribution of gilt position when observed every 15 min versus every 60 min by a chi-square test (R base function). There was no difference in distribution (X^2^ = 12, df = 9, P = 0.2), so we opted for observations every 60 min.

### Personality traits

After the 7 days of free-choice, gilts were moved individually from the experimental pen to a 5.2 × 2.1 m arena with a solid concrete floor to conduct an Open Field/Novel Object (OF/NO) test. Similar to Horback and Parsons^22^, gilts were left to explore the barren area for 5 min, after which a novel object was introduced (50 × 5 cm plastic blue broom head, Vikan, Denmark) and observation continued for another 5 min. Gilts then exited the arena, weighed (mean ± SD: 151.7 kg ± 23.7 kg), and reintegrated into routine farm care. Here, we elected to use OF/NO as the basis of our personality testing. These tests are increasingly employed to quantify traits such as boldness, exploration or fearfulness in many species (for reviews on pigs, see^23,24^).

Behavior observed and their definitions were based on Horback and Parsons^22^ ethogram (see Table 1). During observations, the arena was digitally overlaid with a 3 × 3 grid composed of squares of identical dimension (1.7 × 0.7 m).

**Table 1:** Ethogram used during personality tests (*OF* Open Field, *NO* Novel Object), definitions based on^22^. Times in brackets represent the amount of time for which the behaviour was evaluated (NO: 5 min, OF + NO: 10 min).

Behavior	Test	Definition
Lines crossed	OF + NO (10 min)	Number of gridlines that a gilt’s front two limbs cross. Gilt may be walking forward or backward
Nose wall	OF + NO (10 min)	Duration in seconds the gilt places snout near the walls of the arena. Gilt may be walking or standing still
Nose floor	OF + NO (10 min)	Duration in seconds the gilt places snout near the floor. Gilt may be walking or standing still
Lie down	OF + NO (10 min)	Duration in seconds the gilt lies down
Latency to contact	NO (5 min)	Time in seconds for gilt to make first physical contact with the novel object once it is placed in the arena
Nose object	NO (5 min)	Duration in seconds the gilt places snout near or on the object

### Analysis

Composite traits were extracted from the behaviors observed during the OF and NO tests (Table 1). Behaviors were clustered by Principal Component Analysis (PCA) on R’s FactoMineR package^25^ without rotation. Prior to PCA, the adequacy of the data was examined, ensuring that Bartlett’s test of sphericity was significant (K^2^ = 38.7, P < 0.001) and the Kaiser–Meyer–Olkin measure was acceptable (MSA = 0.61). One gilt did not approach the novel object, which we translated to a *NA* approach latency. We did not exclude this gilt, and conducted the PCA with the *NA* latency, using the *imputePCA* function from the *MissMDA* package^26^. Behaviors with correlations on individual components below − 0.5 or above 0.5 were grouped to elucidate a personality trait represented by that component. Only components with an eigenvalue > 1 were retained for interpretation. Each gilt received a single component score for each trait that was used in subsequent modeling.

Gilt position (in or out of the crate) was analyzed with a logistical mixed model using the *glmer* function from the *lme4* package^27^. Fixed factors were period of day (nighttime: 20h00 to 7h00, daytime: 8h00 to 19h00), experimental day (1 to 7), side of experimental pen (left or right, see Fig. 1) and season (summer: August, 4 trios or winter: November–December, 6 trios). To test for the influence of personality traits on preference, coordinates of each gilt on the components of the PCA were included as covariates (Dimension 1 and Dimension 2, see “Results” section for details). Gilt ID (n = 29: lame sow excluded) was included as a random factor, nested within trio (n = 10). P-values were obtained with the *lmerTest* package^28^ and the significance threshold was set at 0.05 (tendency at 0.10). Odd ratios (OR) and 95% confidence intervals (95CI) were obtained with the *car* package^29^. With 29 gilts observed every hour for 7 days, there were 4872 observations. Due to a failure of the camera system, 2.4% of the videos were lost, bringing the total to 4752 observations.

We assessed individual gilt preference with Markov Chain models to analyze time budget data^30,31^ and evaluate the confidence intervals of times gilts were out of the crate. The analysis considered different durations; the first spanned the whole experimental period over which the study ran, and a more granular approach assessing multiple 24 and 12 h time windows that corresponded to an experimental day and period of day respectively. Data was coded as binary sequences representing gilt location of either 168 in length for the whole experiment, 24 for an experimental day or 12 for an individual daytime or nighttime period. Each animal’s data was fitted to a Markov Chain using Bayesian modelling with the R package *markovchain*^32^ to generate a transition matrix that described the transition probabilities between animal locations. This transition matrix provided the basis to simulate animal location with 10,000 binary sequences of appropriate length (either 168, 24 or 12 elements) using the rmarkovchain function. From the simulated data, variances measures were calculated for the proportion of time individual animal spent out the crate and yielded a 95% confidence interval for each animal-duration combination.

Animals’ preferences were determined based on the expectation that an animal without a preference would spend 71% of their time out of the crate based on the relative space taken by the pen compared to the stalls. Statistical significance was determined by *t*-test at 0.05 significance level.

An additional ordinal logistic regression model (*clm* function from the R package *ordinal*^33^) was conducted to test the predictor significance of Dimensions 1 and 2 from the personality test (both continuous) on the preference of gilts (factor with 3 levels: preference for pen, crate or no preference).

## Results

### Personality traits

The PCA yielded two components (Comp.) for interpretation with eigenvalues for Comp.1 of 2.4, and of 1.4 for Comp.2. These two principal components explained 61.8% of variance in the data (Comp.1: 39.2%, Comp.2: 22.6%). The first dimension was characterized by long latencies to approach the object (r = 0.77), short durations nosing walls (r = − 0.67), few lines crossed (r = − 0.73) and long durations nosing the floor (r = 0.83). We defined Comp.1 as ‘Passivity’. The second component was characterized by short durations of lie or sit (r = − 0.50) and long durations of nosing the object (r = 0.87). We defined Comp.2 as ‘Engagement’. Visualizations of PCA results are presented in supplementary materials (SM1).

### Preference

**Figure 2:**
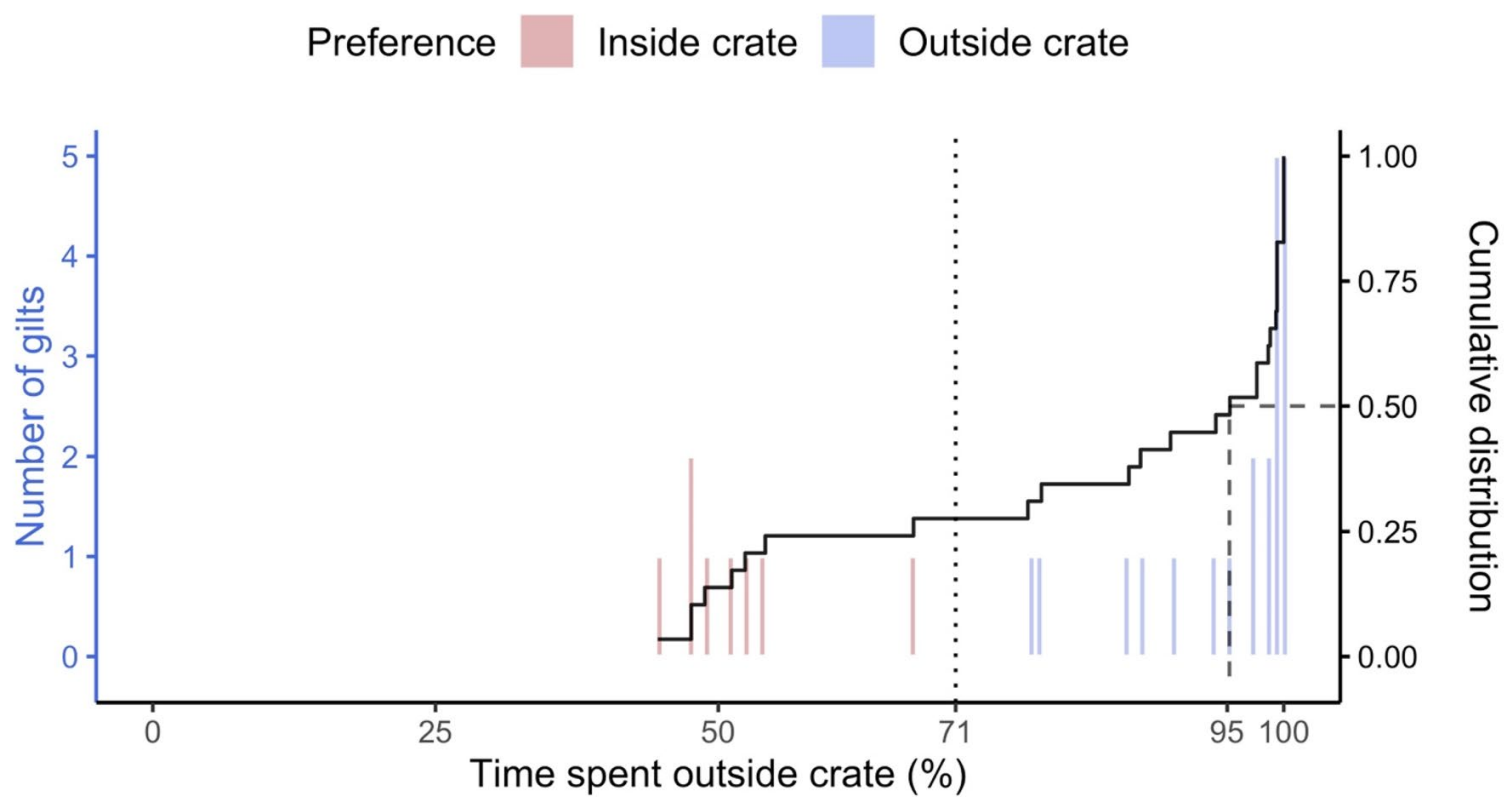
Distributions (raw and cumulative) of the proportions of time gilts (n = 29) spent outside a crate when given free choice with an open-area. Preferences are relative to proportion of space (open area: 71%, dotted vertical line) and tested by *t*-tests (p < 0.05). Most gilts chose to spend the majority of time out of stalls (median = 95.2%, dashed line). Distribution (colored histogram) and cumulative distribution (black steps) of time spent outside of the crate for individual gilts when they had free access to an open area with similar resources (feed and water) for a week. Median time (dotted line) was 95.2% out of the stalls with an interquartile range of 32.1.

All gilts utilized the open area. There were differences in use between individuals in proportion of time spent outside the crates (min. = 44.6%, max. = 100%, Fig. 2). Most gilts spent the majority of their time outside, as the median time outside of the crate was 95.2%. Twenty-one of the 29 the gilts exhibited a significant preference for pen while only 8 preferred the stall over the 7-days trial (p < 0.05). We analyzed 203 gilt-days or 24-h cycles of gilt behavior and found 152 instances where gilts preferred the pen (102 of these gilt days were spent entirely in the open area). Animals were without a preference 8 times, and preferred a crate in 43 instances. Only twice did a gilt spend the whole 24 cycle without leaving the crate.

The first component from the PCA, Passivity, had no significant effect on the proportion of time spent outside (OR = 1.25, 95CI = [0.90, 1.75], P = 0.18), whereas the second component, Engagement, predicted a significant reduction in the time spent outside (OR = 0.39, 95CI = [0.25, 0.60], P < 0.001).

**Figure 3:**
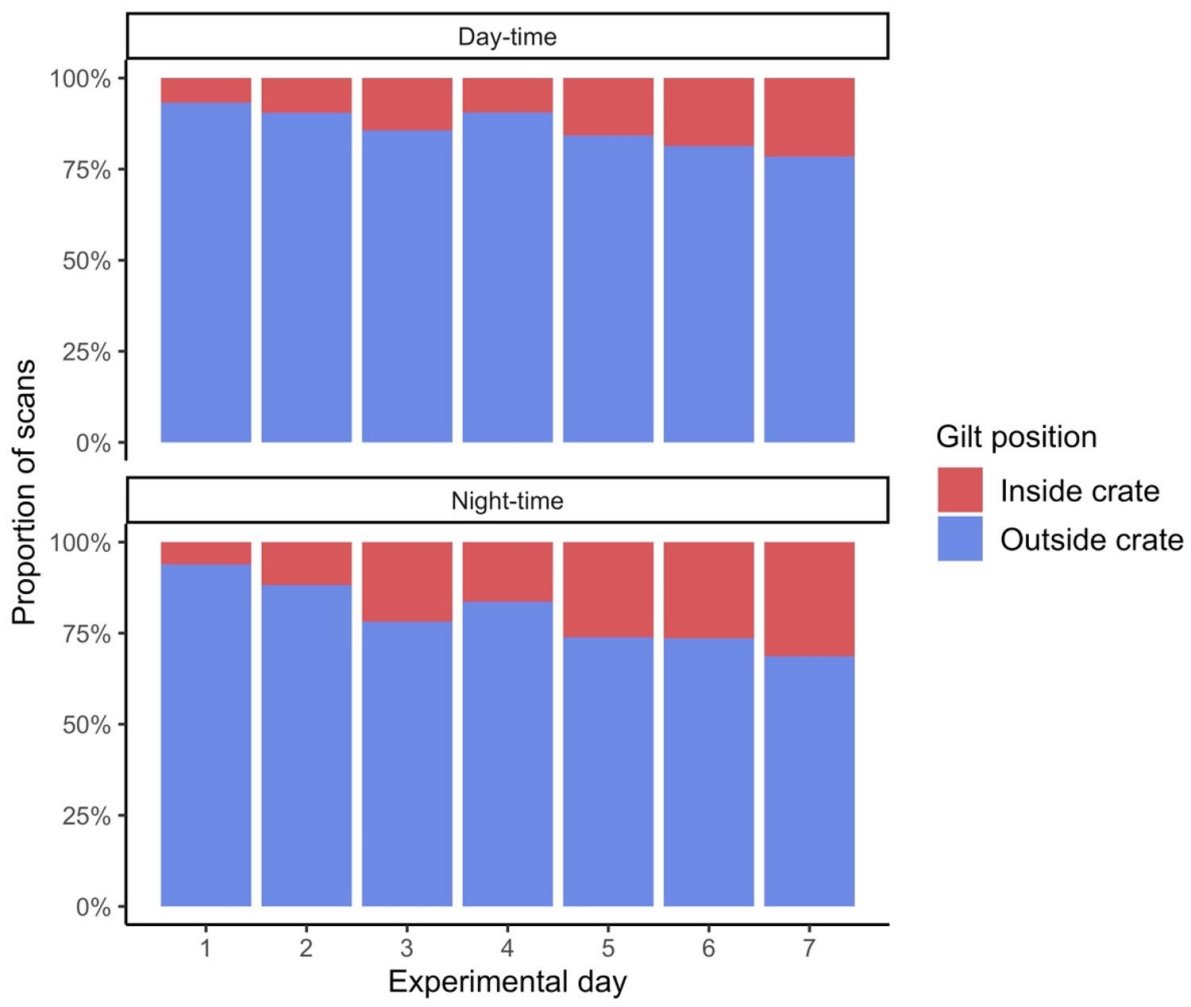
Proportion of scans gilts (n = 29) spent between an open-area and a stall over a week. Gilts reduced time outside of the stall during nighttime and as the trial progressed, despite remaining in the open pen most of the time. Small but significant changes in gilt location were observed by experimental day number (1 to 7) as well as period of day (day vs night).

Gilts also exhibited a significant decrease in their likelihood of utilizing the pen at night (OR = 0.49, 95CI = [0.40, 0.60]) as well as over the duration of the experiment (OR = 0.70, 95CI = [0.66, 0.73], Fig. 3). There was no significant effect of the side of the experimental pen (OR = 0.25, 95CI = [0.02, 3.30], P = 0.29) or season (OR = 0.61, 95CI = [0.04, 9.62], P = 0.72).

**Figure 4:**
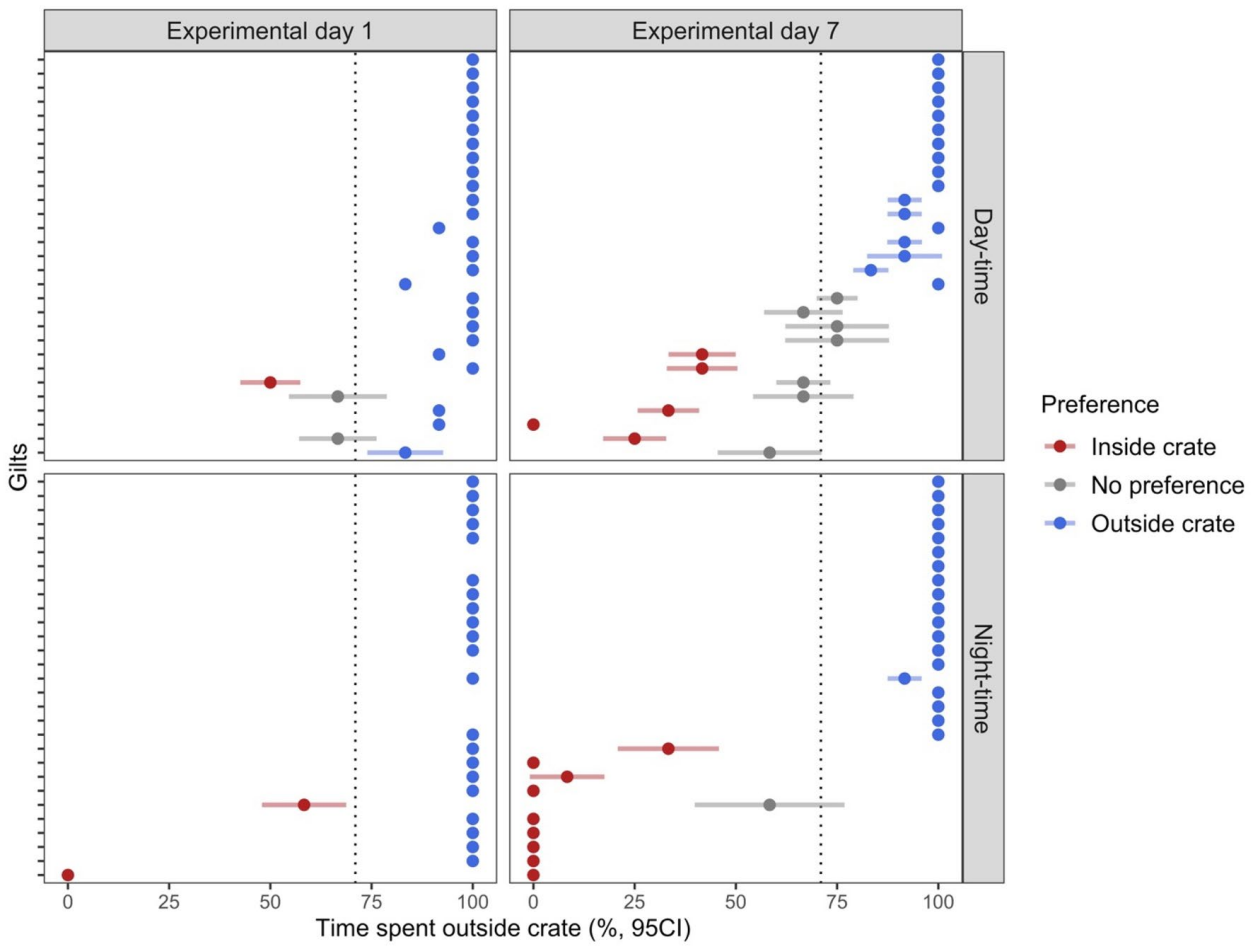
Preferences of gilts (n = 29) for an open-area over a stall when given free-choice. 95% confidence intervals were calculated by Markov Chain models. Preferences are relative to proportion of space (open area: 71%, dotted vertical line) and tested by *t*-tests (p < 0.05). Most gilts prefer to spend most of their time outside of the crates. Gilt behavior does vary depending on period of the day as more animals divide their time between the pen (blue dots) and stalls (red dots) during daytime than at nighttime. A subset of the gilts developed a preference for stalls that increased at nighttime as well as with experimental days. However, most of these animals still utilized both the stall and the pen throughout the day. Grey dots denote an animal without a statistically significant preference. Similar vertical position of data in each of the four quadrants of the figure represents the response of the same animal.

During the first nighttime of the experiment, 27 gilts demonstrated a significant preference for the pen compared to two animals preferring the stall (Fig. 4). By the last nighttime of the experiment, 19 gilts displayed a preference for the open area, 9 displayed a preference for the crate and one animal lost their preference for stall and showed no preference. Gilt behavior, however, was generally more variable during daytime. While 26 gilts preferred the open area during daytime of the first experimental day, exploration of the crate was greater compared to nighttime, as one gilt preferred the crate, 2 showed no preference but 6 of the 26 animals with a significant preference for the pen spent at least 10% of their time in crate. The drive for exploration appeared to increase over the course of the study. By the last day of the trial, 5 of the 19 gilts with a preference for the pens spent at least 10% of their time in a stall. Seven animals demonstrated lack of a preference, and 4 of the 5 animals that preferred the stall still spent at least 25% of their time outside the crate.

Additionally, *Engagement*, but not *Passivity*, also predicted the shifts in individual animal preferences observed on experimental day 7 daytime (z = − 2.01, P = 0.04) and nighttime (z = − 2.0, P = 0.04).

## Discussion

Gilts displayed a clear preference for the open area, with half the animals spending more than 95% of their time outside the crates, and 21 out of the 29 gilts were observed in the open area more often that what would be expected by chance over the 7-day trial. These results fit with previous reports noting that gilts generally prefer short (30 min) over long (4 h) periods of confinement^15^. Our results are also in agreement with studies showing that sows and gilts are motivated to work to exit their stall and access an alleyway for 3 min^18,34^, and that increased space allowance decreases cortisol levels and increases reproductive performances^35^.

However, our results are not consistent with other reports: in a study of free-space usage where sows could access walk-in stalls, sows spent less than 20% (on average) in the free-space, with more than half of the sows spending less than 5% of their time in the open-area^36^. Even with a space allowance per sow higher than in our study, sows only spent around one third of their time in the open area^37^. These strikingly different results from our own were likely caused by differences in methods: larger social groups were studied, sows were of mixed parity, and—most importantly—feed and water were only available in individual stalls, likely explaining the sows’ inclination to remain in the stalls. We argue that previous literature has underestimated pig preference for an open area, and that providing feed and water both inside and outside crates produce more representative data of the animals’ preferences.

It is also possible that our data is itself an underestimation of pigs’ preference for the open area as we only enrolled gilts, who might be less bothered by the crates because of their smaller size. As previously noted, age and pregnancy status likely drive the usage of free-space, with heavier sows using an open area more than lighter sows^36^.

The observed decrease in preference for the open area with experimental days could be due to an habituation effect^38^. During the daytime on the first day, 20 animals were observed only in the pen while 9 explored the crate, whereas by the last day 17 gilts were spending at least some time in a crate. Thus, as time goes by, gilts perhaps become accustomed to the open area and their interest to explore the lesser-known crate grows. This explanation seems probable, as the open area was barren, hence unlikely to elicit extended interest from the gilts. Another reason, perhaps in combination with the previous one, would be that gilts lose their initial aversion to the crates as objects of space restriction when they understand the possibility to move freely in and out of them. By the last daytime, most gilts were using both the stalls and the pen, with 7 of them showing no particular preference. Interestingly, five animals never overcame their initial aversion to the stalls and spent 100% of their time in the open area.

As we focused our study on gilts’ preference over only seven days, it is unknown whether the decrease in use of the open area would have continued or if it had reached a plateau. Rioja-Lang et al.^36^ did not find differences in free-space usage over several weeks, but studies on swine long-term preferences remain scarce.

The cause for gilts spending more time in crates during night in comparison to daytime remains unclear. Fraser^11^ noted that pigs’ bedding preferences were dependent on ambient temperature, so it is possible that cooler night temperatures made gilts more likely to be in crates. However, as we did not observe an effect of season, it is unlikely for temperature to be an overriding factor in gilts’ crate usage. Perhaps crates felt innately more secure when visibility was limited due to the fewer possible entry points, similar to a nest or burrow. This remains speculative.

The relationship between preference data and personality traits was defined using behavioral responses to an OF/NO test. The trait we interpreted as *Passivity* was not found to have a relationship with time spent outside the crate. However, the trait characterized as *Engagement* had a significant relationship, with more engaged gilts spending increased time inside crates. These findings could appear counterintuitive, but in a study of preference between lengths of confinement in crates^15^, individual differences were also found, with some gilts even preferring long over short confinement. Authors mentioned that the interest for an apparent aversive environment (space restriction of a crate) could be an exploratory or monitoring behavior, allowing them to survey their surroundings^39^. This interpretation could fit with our results that gilts who were more engaged with exploration during an OF/NO test were more likely to investigate the crate.

There are a couple of possible limitations in our understanding the role of individual differences in housing preferences. First, in this study the definition of individual differences was somewhat rudimentary as only an OF/NO test was employed. A more extensive battery of tests, as used in the past^22,40^, might yield additional behavioral traits that also could be predictive of housing preference beyond *Engagement*. Second, life history of these gilts is not totally known. Although we attempted to homogenize their experience once they were under our care (at approximately 24 weeks old), the influence of previous experiences, such as human interactions, housing, and cognitive enrichments on responses in a personality test have been noted^41–43^. We only studied crate-naive gilts, but an interesting avenue of research would be the influence of previous experiences with crates on their usage. Although research has reported that sows from either group-housing or from gestation stalls displayed a strong preference for unlocked over locked stalls^16^, potential effects of previous confinement on preference tests have been noted^8^.

Repeating personality tests is often mentioned as a way to validate the reliability and robustness of the test, ensuring that the traits observed are consistent through time which is integral to the definition of personality^23^. We chose to conduct a single personality test because the validity of repeated novel tests (environment or object) has been questioned, in favor of results from the first test^44^. A recent review has also reported the low repeatability of novel object tests in pigs, suggesting their quick adaptability to novelty^23^.

Our present study highlights the importance of access to an open area for gilts as less than 1% of the time did any gilt spend a 24 h cycle without leaving a crate. While a subset of the animals exhibited a preference for a crate, 99% of the time animals still utilized the ability to access open space. Our work provided a relatively barren open area, but future studies could investigate how more complex environments including enrichment, substrates, and different social dynamics impact housing preference. Preference studies also need to be complemented by investigations into the motivations to access or escape different conditions. Although gilts preferred the open area, they might still highly value the opportunity to occasionally isolate from conspecifics, especially if animals are unfamiliar and mixed within a restricted space^45^.

## Conclusion

Gilts displayed a clear preference for an open area when given the choice between a crate and a pen. Although individual differences were observed, half the gilts spent more than 95% of their time outside the crates and 21/29 of the animals expressed a preference for the open area. A small subpopulation of animals developed a preference for the stalls but still utilized both the stall and the pen throughout the day. Time of day and experimental duration affected gilt behavior as animals spent more time in the crates at night and as experimental days passed. Personality traits also influenced gilt preference with more *Engaged* gilts during a OF/NO test spending more time in crates.

This study compliments a body of research indicating that restricting pigs to crates is likely experienced as negative and has the potential to compromise their welfare.

### Supplementary Information

Supplementary Information 1.

Supplementary Information 2.

Supplementary Information 3.

Supplementary Information 4.

## Data Availability

Data and R code are available in supplemental materials.
