# Supplementary material for: Gilts prefer an open pen to a stall
Source: Sci Rep. 2024 Apr 27;14:9684. doi: 10.1038/s41598-024-60617-2 (PMC11055906; doi:10.1038/s41598-024-60617-2)
Supplement: Supplementary file 4 — Supplementary Information 4. [file 41598_2024_60617_MOESM4_ESM.docx]

**Supplementary material – details on Principal Component Analysis (PCA)**


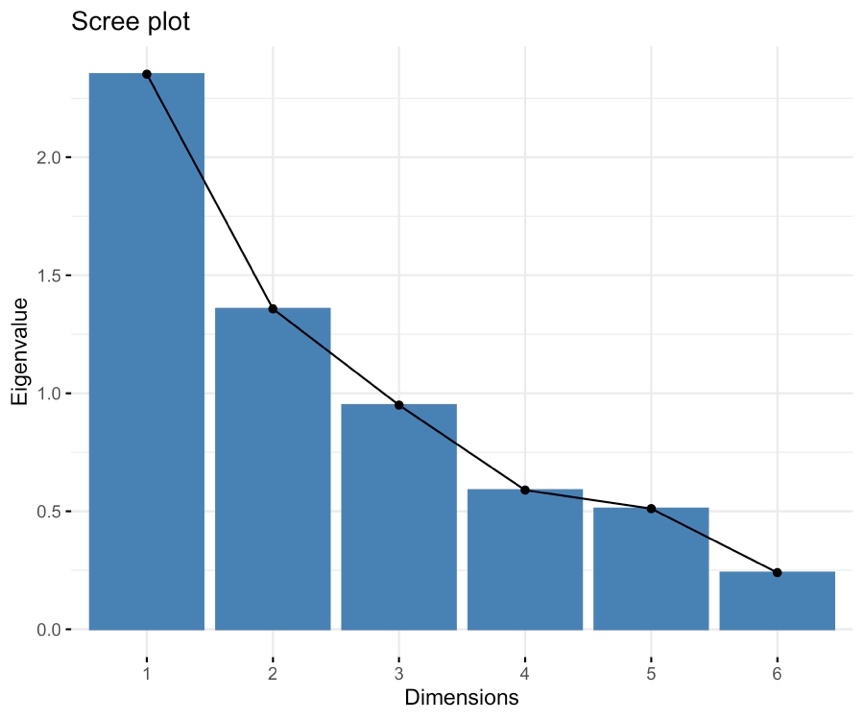


***Scree plot.*** *Eigenvalues of the first 6 dimensions of the PCA. Dimensions with an eigenvalue above one were selected for further analysis*


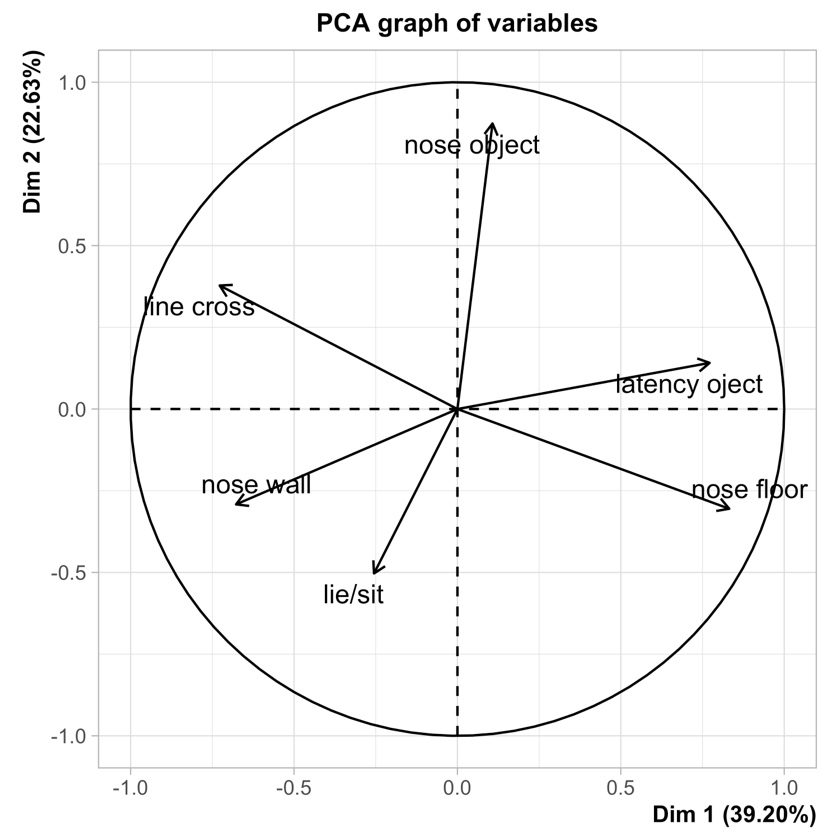


***PCA visualisation.*** *Projection of behavioral variables observed during an Open Field/Novel Object test on the two principal components with an eigenvalue > 1.*


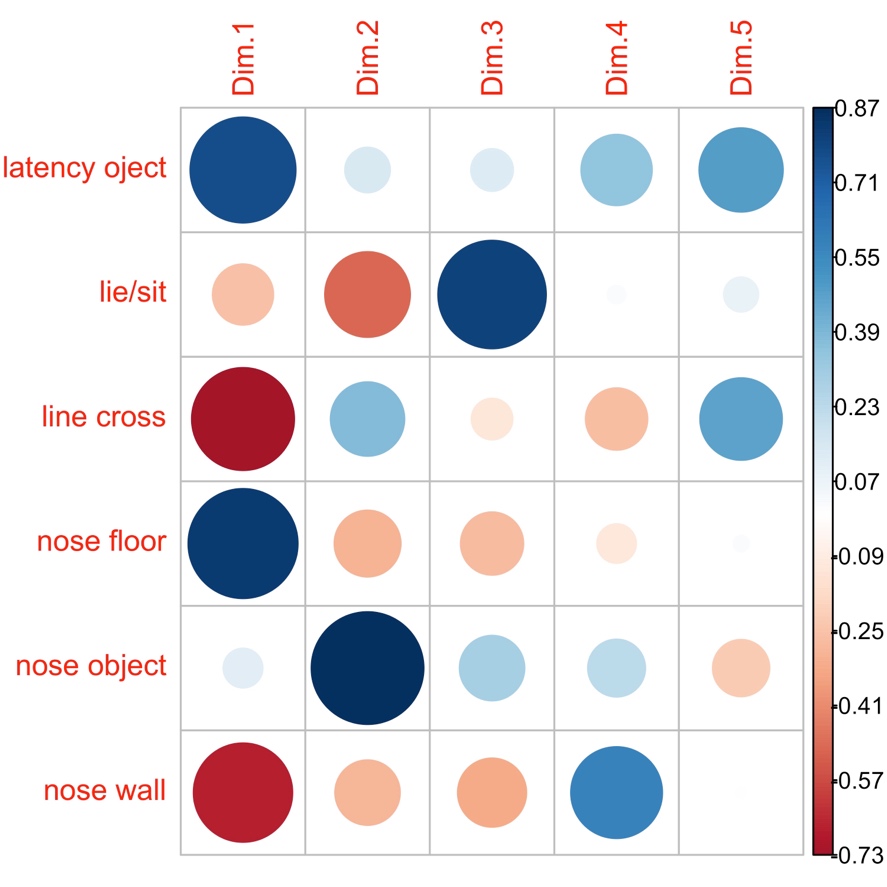


***Correlation matrix.*** *Detail on the correlation of each variable on principal components. Blue represents a positive correlation, red a negative one. The size of circles is proportional to the strength of the correlation.*
